# Collectanea

**Published:** 1832-04

**Authors:** 


### COLLECTANEA.

Floriferis ut apes in saltibus omnia libant,
Omnia nos, itidem, depascimur aurea dicta.

### PHYSIOLOGY.

### *Discovery of an Anastomosis between the Fourth and Fifth Pair of Nerves.* By Mr. Fearn, of Edinburgh.

This interesting contribution to physiology was published in the Lancet, No. 445. This anastomosis was discovered by Mr. Fearn last winter, whilst dissecting for Dr. Macartney, of Dublin: he states that the communication commonly takes place by means of a slender twig, which proceeds obliquely upwards and forwards, from the first division of the fifth to the fourth pair, as these nerves are passing the cavernous sinus on their way to the orbit, and immediately before the fourth pair comes to be in contact with the first division of the fifth. Occasionally, however, the anastomosis occurs in a manner somewhat different; and this is the case in a preparation in Mr. F.'s possession. The filament, instead of taking the course above mentioned, passes in a direction obliquely from behind forwards, from the fourth pair to the first division of the fifth. The dissection in which this discovery was made by Mr. Fearn, was very closely examined with a lens by Dr. Macartney and many others in Dublin; and from the evident plexiform arrangement of the fibres of the respective trunks with the anastomosing filament, no doubt whatever was entertained of the communication.

Mr. Fearn, whilst in Edinburgh, shewed a fresh dissection to Dr. R. C. Rudolphi, of Berlin, Mr. Lizars, and other eminent anatomists; and they were all equally satisfied of the existence of the anastomosis. As there is no mention made of it in the works of Soemmering, Scarpa, Sevan, or any of those who have made minute anatomy their particular study, Mr. Fearn infers that it has hitherto been unknown to the profession.*

### PATHOLOGY.

### *Hydrophobia, from the Bite of a Cat, occurring three months after the Injury.*

This case is related in the Bull. des Sciences Med. for July 1831 We do not deem it necessary to give the whole of the original details. The fact of so unusual a length of time having elapsed between the bite of the animal and the commencement of the hydrophobic symptoms, is too important to be passed over. A man, thirty-two years old, was bitten on the lower lip by a young cat that he was endeavouring to carry away. The wound was trifling, but bled a good deal; the patient attached no importance to the accident. His health continued good until about ten weeks afterwards, when he felt a slightly unpleasant roughness and oppression in the throat. He had entirely forgotten the circumstance of his having been bitten, when, on September 5th, (three months after the bite had been inflicted,) as he was about to drink some wine with a comrade, he felt a dislike to the liquid, and a slight spasm, which much surprised him. He returned home, and the next morning the well-marked symptoms of hydrophobia existed, and gradually became more severe: he died in the Hôtel Dieu.

* A correspondent in a subsequent number of the Lancet states, that this connexion between the fourth and fifth nerves has been mentioned before by some writers.—Ed.

### *Cases of Hydatids of the Uterus, simulating Pregnancy.*

By JOHN ANDREW, M.D.

CASE I. April 3, 1815. Miss M. R., aged sixteen years, has been in a bad state of health for the last six months, during which time the catamenia have ceased, and the abdomen is rapidly increasing in size, and, at present, she is as large as a woman in the eighth month of pregnancy. The abdomen is prominent, and hard to the touch; the mammæ are enlarged and firm, with a copper coloured areola surrounding the nipples. She has been spitting blood, occasionally, for the last three weeks, but which, owing to the upbraiding of friends and others, she has hitherto concealed. She is very much depressed in spirits, and, to use her own language, she wishes death rather than life, that she may be freed from the taunts of her relations. There is slight cough, with occasional flushings of face. Pulse 102, and soft. Tongue white. Bowels costive. Urine in natural quantity.—℞. Ol. Ricini ℥i; patient to be strictly watched.

4th. Castor oil has operated well, but she says she has experienced no relief. Has spit up about a teaspoonful of blood during the night. Pulse eighty-three, and soft. Tongue whitish. Urine in natural quantity.

5th. Three o'clock A.M. I was summoned, in haste, to attend Miss M. R., who had cut her throat with a penknife, and wounded the labial artery and trachea. The artery was tied, the wound dressed, a strait-jacket put on, and a proper person appointed to take care of her.

Eight o'clock A.M. I am informed that after leaving her, she slept till about seven o'clock, when the nurse, at her request, took off the strait-jacket. Determined on self-destruction, she tore off the dressings, and attempted to tear away the ligature, since which time, she has a short tickling cough, while a quantity of bloody mucus has been discharged from the opening in trachea. Pulse eighty-four, hard. Tongue white. Eighteen ounces of blood have been taken from the arm, with relief.

Nine o'clock P.M. She is now more composed, and has slept about an hour. At present she complains of pain in the head and stiffness about the neck. Pulse ninety-three, soft.—Head to be shaved and bathed with vinegar.

6th. Nine A.M. She has no complaint but a slight pain in the abdomen, which comes on at irregular intervals. She has slight cough.—℞. Tinct. Hyoscyami nig. ʒi; Tr. Digitalis gtts. xx.; Aquæ Puræ ℥i. Fiat haustus statim sumendus, et reptr. H. S.

Wound was dressed on the 8th, 9th, and 10th, when the ligature came away, and it is slowly healing.

11th. Five A.M. Has complained of irregular pain in the abdomen since one o'clock this morning, which, within the last hour, has been accompanied with a degree of pressing down. Upon examining per vaginam, the os uteri is found dilated to the size of a shilling, and there is a glairy discharge from the vagina. Pulse ninety: bowels costive.—℞. Ol. Ricini ℥i.

Nine A.M. Castor oil has operated three times, but the pain has become worse within this half hour. Examining per vaginam, there is an elastic tumor found protruding without the os uteri.

There was discharged, about eleven o'clock, a bag about the size of a new-born child's head, which contained a clear fluid resembling water; and an hour afterwards, a large mass of vesicles was discharged, followed by considerable hemorrhage. A compress and bandage have been applied, patient's head and shoulders have been lowered, and rest enjoined.

Two P.M. Another mass of vesicles has been discharged, resembling the former. The hemorrhage continues. On passing the hand into the uterus, it is found dilated and relaxed, but on moving the hand about, the uterus freely contracts around it, and the hand is expelled, along with some clots of blood. The bandage was tightened, and the patient left at rest. After this day she continued to improve. The wound in the throat soon cicatrised; she gradually recovered her health and spirits, and by the 24th April she was quite well.

CASE II. September 28th, 1831. Mrs. F—, a sailor's wife, has been spitting blood for the last ten days. She complains of pain in the breast, tickling cough, and an inclination to vomit in the mornings. She has been married about twenty-two months, and had an abortion three months after. She says that the catamenia have not appeared for the last four months, and that she thinks she is pregnant. The abdomen is prominent and firm to the touch; she is larger in appearance than a woman in the sixth month of pregnancy. Pulse eighty-four, and hard; bowels costive. Twenty ounces of blood were taken from the arm, and a dose of castor oil administered.

29th. Since the operation of the castor oil she is much relieved in breathing and pain in the chest. She says that the pain in the abdomen is increased, and that it is now more like the pain she experienced when she had the abortion some months ago. Spitting of blood still continues. Pulse eighty, and soft.—℞. Tinct. Hyoscyami nig. ʒi.; Tr. Digitalis m. xx. Syrup. simpl. Aq. Cinnamom. *aa.* ℥ss. Capiat quamprimam, et reptr. H. S.

30th. She says that she had a good night's sleep till about five o'clock this morning, when the pains became more severe, and accompanied with hemorrhage. Examining per vaginam, the os uteri is dilated to about the size of a half-crown, and there is an elastic tumor protruding. In the course of the forenoon there was discharged from the uterus a large bag containing a clear fluid like water, but without any fœtus contained in it; and soon after, a large mass of vesicles of different sizes and shapes, weighing about seven pounds, and containing a glairy fluid, came away. There was still no appearance of fœtus or placenta. On introducing the hand into the uterus, the contents seemed to be evacuated.—Reptr Haustus.

October 2d. Appears to be doing well, but she has spit up a small quantity of blood during the night, not more than the fourth part of a teaspoonful. The pain and hemorrhage have ceased. Pulse eighty-four, and soft; tongue white; bowels costive.—℞. Ol. Ricini ℥i.

After the 2d, she had no return of the hæmoptysis, and rapidly recovered her health and strength.

CASE III. December 26th, 1830. Mrs. S. wife of a respectable tradesman, has been married above six months. She says that, for the last four months, the catamenia have not appeared, and that from her appearance and symptoms she has every reason to believe that she is pregnant. For the last eight days she has had violent cough, which is unattended with pain in the chest. The cough is very troublesome, when she attempts to lie down in bed. Last night and this morning, she has spit up a small quantity of blood. Pulse ninety, and soft; tongue white; bowels costive. ℞. Ol. Ricini ℥i. Habt. mistur. pro tussi.

27th. She says that the cough is not so frequent or severe as it was yesterday. Has spit up no more blood. Pulse ninety.—Cough mixture to be continued.

28th. This morning has spit up about a teaspoonful of blood. She complains of irregular pains in the abdomen, which come on at intervals. The abdomen is prominent and firm. She says that she has sickness in the morning, and the appetite is capricious. Her breasts are enlarged and prominent, and the areola around the nipples is distinctly marked.—She was bled; rest enjoined, and an opiate administered.

29th, seven A.M. Since two o'clock this morning the pains have returned with more severity, and there is at present considerable hemorrhage. On examination per vaginam, the os uteri is dilated to the extent of a halfcrown piece, tender and hard when touched. There is a substance of a doughy feel protruding, devoid of that elasticity which is felt when the membranes containing the liquor amnii protrude. The pains continued every three minutes, till about ten o'clock, when there was expelled from the uterus a fleshy mass resembling the placenta, with a number of small vesicles on the surface, but there was no appearance of either umbilical cord or fœtus. Examining again per vaginam, an elastic bag was felt protruding at the os uteri, and the pains continuing regular, with pressing down, it was soon discharged entire. On opening it, it was found to contain a fluid resembling the liquor amnii, but no appearance of a fœtus. A short time after, a large quantity of vesicles were discharged, of irregular size and shape, from that of a pea to that of a large hen's egg; they contained a glairy fluid. In about half an hour after there was a quantity more discharged, the whole, when collected filling a handbasin. As there was considerable hemorrhage, and the uterus not contracting, I introduced my hand into the uterus, at the same time pressure being made on the abdomen, I ascertained that a portion of the same mass was contained there. The uterine action now became so severe and continued, that I was able to detach but a very small portion of them. The hand was withdrawn, a compress was laid over the uterus, and bandage was applied round the body, upon which pain and hemorrhage ceased.

Four P.M. No return of pain or hemorrhage; she says, that she now feels comfortable.

30th, nine A.M. The pain has returned within the last hour, with bearing down, and there is hemorrhage. Examining per vaginam, the os uteri is found dilated so as to admit the hand, and there is a portion of the mass protruding.

Ten A.M. There is just discharged nearly as much of the same substance as was discharged yesterday. On introducing the hand into the uterus, in order to excite the uterine action, I ascertained that the whole was discharged. She has spit up a little more blood this morning. On examining carefully what was discharged from the uterus, no fœtus can be discovered.—Cough mixture to be continued.

The cough and slight hemorrhage continued for a few days, but by 7th January she was able to be out of bed, and soon after recovered perfectly.

Case IV. July 4th, 1831, two A.M. Miss S. H., aged seventeen years. Her mother informs me that she has enjoyed good health until within these last five or six months, when she began to look pale, complained of a pain in the head and back, accompanied with sickness and occasional vomiting. For the last thirteen weeks she has been observed to swell, but the swelling has been very rapid during the last month, so that now she is as large as a woman in the seventh month of pregnancy. The abdomen is prominent and firm, the breasts are swollen, with an areola of a slight copperish colour around the nipples. She has occasionally spit up blood. Dr. D. Anderson, her medical attendant, has treated her with diuretics and laxatives, on the supposition that she has dropsy of the right ovarium.

Since twelve o'clock last night, she has been attacked with severe irregular pains in the abdomen. The bowels are rather loose, and there is tenesmus when at stool. As the catamenia had not appeared, I proposed an examination per vaginam, in order to ascertain if the catamenia were not retained. I found the hymen entire, with a small aperture in the centre. I cautiously introduced my finger into the vagina. I could not feel the os uteri, although the body of the uterus was distinctly felt, enlarged and of the same form as in the state of pregnancy. An anodyne draught was administered.

Three P.M. I was called in haste to visit my patient, who had become convulsed. She is at present comatose, and when aroused she talks incoherently. The pains are now severe and irregular. Thirty-three ounces of blood were taken from the arm, which gave relief. Anodyne draught to be repeated. The pains, however, became regular and pressing down as in labour, and the os uteri was soon dilated to the size of a halfcrown piece, and an elastic tumor was felt projecting. In the afternoon a large bag was protruded at the os externum, which was found to contain a glairy fluid, not unlike the white of an egg. In a short time there was again discharged a mass of vesicles, containing a similar fluid. On passing my hand into the uterus, I found there was a quantity more, which was expelled along with the hand. Considerable flooding followed, which was restrained by a compress and bandage, and steady pressure; feels faint; pulse sixty-five, feeble. After the discharge of a small portion more, the uterus contracted, and she fell asleep, the pulse gradually rising.

5th. Has been very restless since she awoke about half-past eleven o'clock last night; complains of slight pain in the abdomen; skin hot and dry; pulse 110; tongue dry; great thirst.—℞. Ol. Ricini ℥ss. Ol. Terebinth. ʒii. Aq. Cinnam. ℥i. Aquæ Potassæ gtts. viii. M. Capiat quam primum.

Medicine operated well, with relief to all the symptoms. She continued after the 6th daily to gain strength, and in a short time got quite well.

Now let us inquire what are the characteristic appearances of a female who has been recently delivered. A ruggedness of the internal coat of the uterus, the increased size of its cavity, the thickness of its parietes, the large size of its mouth, admitting two or three fingers, the sanguineous discharge, the relaxation of the external parts, their colour, the prominence of the abdomen, the relaxed and wrinkled state of the integuments, the enlargement of the mammæ, and those containing milk. These are the appearances characteristic of recent delivery, and these were all present in the above cases, and therefore the whole of them may exist independent of delivery.

Had both of the young ladies, whose characters, I have no doubt, were unimpeachable, died from peritonitis, convulsions, or hemorrhage, and a legal investigation taken place, what would have been the medical report? Doubtless, that the diseased had died in childbed, there being evident appearances of a recent delivery; and the reporters not knowing the history of the case, would be borne out in their opinion by gentlemen of integrity and observation. Where we have not the existence of a fœtus, a placenta, or the confession of the female herself to form our judgment by, ought we not to be extremely cautious in giving our evidence in a court of justice?

I have no hesitation in saying, that there ought not only to be a placenta, but a chord attached, or the appearance of that being torn away from it, before a medical gentleman should say that there has been a recent delivery.—*Glasgow Medical Journal.*

---

### *Cases of Asphyxia.* By Henry Ewen, Esq.

About ten o'clock in the morning of Monday, January 30th, I was called to visit two men at Sutton Bridge, who were supposed to be suffocated on board the brig Economy. I found them supported on chairs in the kitchen of the Bridge Hotel. They were completely insensible; their countenances were a livid purple; the extremities and surface generally cold; pulse weak, irregular, and frequent, scarcely to be distinguished; breathing stertorous; jaws firmly clenched.

I directed them to be placed in the recumbent posture on a mattress, before a good fire, the head and shoulders being raised, and supported by pillows. Bottles of hot water were placed on each side of their trunks and between the thighs; diligent frictions were applied to the extremities, and sinapisms to the soles of their feet. I next introduced an elastic tube into the stomach, and forcing open the jaws with the handle of a common table-fork, injected some hot brandy and water, the beneficial effect of which was obvious to all. After an hour this was repeated, when the pulse became stronger and regular, but was very frequent, (120 a minute.) The face became of a natural colour, the lips being red; the breathing also was less laborious. One of the patients vomited freely after injecting the warm fluid into his stomach; the other had done so on being first brought out of the cabin on deck. The food ejected consisted of meat, &c. quite unchanged, which had been taken for supper the preceding evening. Friction and the application of warmth were continued till the circulation of the blood was fully restored. Blood was abstracted from the arm after the hot brandy and water was exhibited, with a view of relieving the congestion of the brain and lungs. The patients were then placed in warm beds. One had an involuntary evacuation of fæces, the other retention of urine; the former shewed signs of returning sensibility towards evening, and took a pint of boiled sago. He was still in a state of stupor, but capable of being roused by speaking loud and sharply to him. The next morning this man was quite sensible, and only complained of extreme weakness, (being incapable of raising his head from the pillow,) and of "great soreness" all over him. He progressively recovered; his pulse, in the course of twenty-four hours, returning to the natural standard. The other man recovered much more slowly; sensibility returning the following evening, not until thirty-six hours after he was removed from his perilous situation. It was necessary to introduce the catheter several times. I injected a camphor enema into the rectum, applied twelve leeches to his temples, and a blister to the nape of the neck. This man also was very weak for two or three days, and complained of "great soreness all over" him. They expressed themselves, on becoming sensible, utterly unconscious of any thing which had occurred from the time of getting into their hammocks on Sunday evening.

It may be right here to state, that the men had had a fire in the hold on the preceding day, which was left burning with the hatches fastened down.—*Med. Gazette.*

---

*Insanity alleviated by Acute Diseases.* (From *Observations on the Medical Treatment of Insanity*, by Dr. SEYMOUR.)

IT occurred to me, when visiting, many years ago, the asylum for lunatics at Charenton, near Paris, to ask the superintendant whether the presence of acute diseases suspended, or altogether removed, the mental distress. The answer was, that they had had many examples of it; patients having become perfectly sane during a severe attack of fever, and some few appeared to have recovered their senses, others to have relapsed. Esquirol states that he has observed this salutary influence of the law, which seems to effect the suspension of a chronic by the presence of an acute disease. The occurrence of intermittent fever, especially of a quartan type, is considered by many observers of a particularly favorable nature. The following cases rest on the testimony of M. GUISLAIN, physician to the Asylum for Lunatics at Ghent:

M. Boatman, aged forty years, of an athletic temperament, has been for several years at the hospital for lunatics at Ghent. He became maniacal in consequence of violent fright: one day an endeavour was made to seize his person, to impress him into the naval service, but he fought with such spirit and success that he escaped from the gens d'armes. Some days afterwards he lost the use of his reason, and mania appeared: he was transferred to the asylum. The patient passed two years in that state, and was much feared by his companions in misfortune, on account of his great strength. At the end of this time, and in the early days of November, he was attacked with fever: the fever returned daily with great regularity, and with considerable violence, during two months. At the end of this time the febrile condition diminished, became irregular, and at length ceased completely. A sensible amelioration of the mind immediately followed: the patient became extremely docile; no violent passion was now remarked; still he was not perfectly sane, there still remained some incoherence of ideas; but, if I may judge from the present state of the disease, his recovery will not be slow.

The next is the case where continued, or typhoid fever, produced a fortunate change in the mind of a maniac furiously mad.

A Prussian soldier received corporeal punishment for a crime committed in his regiment, and from that time mental derangement ensued. He was transported to the military hospital at Ghent, where, after having been only disturbed in his mind, he suddenly became furiously mad: he subsequently passed eighteen months in a condition completely passive, without the slightest emotion, and without the smallest inclination. An excessive fear was the predominant character of the disease. He was attacked with typhous fever. The period of invasion was soon passed, and was succeeded by an extreme prostration of strength: black tongue, sordes on the lips, livid countenance, involuntary dejections, meteorism, low delirium, and other symptoms of a similar character, marked the height of the malady. Towards the twentieth day, the patient presented a more expressive countenance; he lay upon his side, and gave reasonable answers to questions for the first time. He inquired about his condition, the place where he found himself, and other circumstances which related to him. For some weeks the convalescence was complete, and he left the hospital without the smallest trace of mental disease.

It is extremely difficult to reason on these very remarkable cases. With regard to intermittent fever, however, it may be doubted whether such attacks are true ague, or really symptoms of change in the nervous disease itself. Many organic diseases, and none more particularly than those of the brain, shew a tendency in their symptoms to become intermittent. Intermittent headach is often accompanied by noises in the head, and great trembling and distress; but such intermission is an encouraging circumstance, viewed in reference to the recovery of the patient. My own experience does not enable me to determine this question; but I suggest it for the consideration of those who may have opportunities of observation, whether the intermission is not the result of a change in the sensibility of the brain, arising in the progress of cure, rather than a new disease, occupying the seat of a disease of a totally different kind. Such an explanation cannot, however, apply to the cases in which pulmonary consumption has been seen to be arrested by the presence of mania. Dr. Mead's case is one of the most striking examples, and I prefer it because the authority on which it rests is indisputable. He relates that a young woman was attacked with hæmoptysis, for which she was repeatedly bled; but, although the symptoms were relieved, the disease was not cured. In two months, hectic fever supervened, with short cough, thirst, heat, and nocturnal sweats: these were accompanied with great emaciation, and the excretion of puriform matter. True phthisis pulmonalis was present, and death at the very threshold. The patient was desirous of the offices of religion ; but it appears she was greatly alarmed by the vivid picture of punishment in another world for unrepentant sinners, presented to her view by the clergy : at least, Dr. Mead speaks of their conduct in no measured terms of disapprobation. The consequence was, that the unhappy patient became the victim of religious mania; by night and day, demons and flames presented themselves to her imagination, and hell appeared open before her. From the invasion of this horrible mania, the symptoms of consumption began to decline, the febrile heat was diminished, the expectoration restrained, and the whole aspect of the disease was such, that the body appeared to grow more healthy just in proportion as the mental powers became more injured. A few days after the madness disappeared, the phthisical symptoms returned, and in about three months the patient expired.

It has occurred to me, more than once, to see similar cases; and I more particularly remember one, where, during three months, the return of the maniacal symptoms was accompanied by an entire suppression of the expectoration, when a new vomica appeared to give way, and the patient's mind became clear and collected, without a trace of the delusion which had previously obscured it.

Frank relates a case where the incidents occurred inversely. A lady, predisposed hereditarily to mania, presented all the symptoms of mental derangement, accompanied by sadness and disgust for life; hæmoptysis ensued, and at the same time the intellectual trouble ceased. The two diseases succeeded one another a second time, and the patient was completely cured.

---

*Some additional Remarks on Permanent Spasmodic Stricture of the Great Intestine; its Appearances and Treatment.* By JOHN HOWSHIP, Surgeon to the St. George s Infirmary, and Teacher of Surgery.

IN a little essay upon spasmodic stricture in the colon, lately published, it was my object to point out the occasional occurrence of spasmodic contraction, or stricture, in the great intestine; to shew that this stricture frequently gives rise to all the inconveniences induced by habitual confinement of the bowels; and to demonstrate that there is a mode of treatment by which this complaint may be ascertained, and by which it may also be removed.

I there ventured to state (what I believe to be true), that the operation of injecting a fluid into the great intestine, for the specific purpose of first determining the existence of spasmodic stricture, and then relieving and removing the contraction, is an entirely new proposition in surgery; and that although from what I have seen of its effects, I have the utmost confidence in its powers as a successful means, that confidence is as yet rather the effect of close than the result of very extensive observation. I still continue, therefore, carefully to collect every new fact upon this subject; and hope that by this course, aided by the kind assistance of some of my professional friends, I may, in a few years, be enabled to bring the more important points regarding the symptoms, diagnosis, and treatment, into a comparatively distinct and satisfactory form.

That the bowels are occasionally subject to painful spasm, has been long known; but as the pain in such cases has made the strongest appeal to the feelings of the patient, and the attention of the practitioner, it has been generally, if not invariably believed, that the pain and the spasm were inseparable companions; a conclusion which I have found to be entirely at variance with the truth.

As to pain, presumably in the bowels, it has usually been considered impossible to come to any precise or clear conclusion with regard to its exact situation; but in the essay mentioned, it was shewn that where certain affections productive of uneasiness, pain, or costiveness, are situated in any part of the large intestine, the intelligent practitioner may always determine the affected part with precision; and that in the great majority of such cases, were these complaints attended to (as they ought to be) in their commencement, we should much less frequently see mere disorder induce disease.

I have there demonstrated, among other points, the astonishing extent to which acute febrile excitement, with severe local pain, may go in hurrying the case on to a fatal conclusion, which no medical means can avert; when the simple expedient of distending and washing out the painfully contracted and irritable colon relieved, and at once removed, all the symptoms in a manner scarcely to be credited. In short, the most complicated train of evils, connected as so many links of the same chain, are sometimes induced by complaints originating in the great intestine, exciting in the first instance either local pain or permanent spasm, or perhaps both.

Without adverting further to what has been before advanced, I shall now mention some particulars of a case in which the seat of spasm (by the adoption of the appropriate treatment) was first deprived of its painful character, and then relaxed; although it is curious that it did not recover its susceptibility to the usual griping uneasiness excited in other parts of the bowel under the influence of distention. I shall, then, in conclusion, describe the peculiar characters of a preparation I lately made, demonstrative of the precise appearances of this form of stricture, as also its induced consequences during life.

CASE. August 3, 1830. Mary Cape, æt. thirty-three, had been a month under my care, in the St. George's Infirmary, for pain in the left side, with a sense of soreness. I applied a blister twice, and with benefit, while they remained open. She said that four years since she had been confined for seven weeks with nervous fever; and that the bowels, always regular before, had never since acted at all, except when she took medicines for that purpose. It appeared, however, from her account, that as long as nine years since she was kicked by her husband while pregnant. This injury was in the left side, between the lower ribs and the margin of the ileum. A premature stillbirth followed, and from that time she had occasionally felt an aching uneasy pain in the apparent seat of the sigmoid flexure of the colon. Since the nervous fever (she said) this pain, in the same part, became more constant and more considerable, and she thought induced the habitual confinement of bowels which then followed.

It was, as she expressed it, an irksome aching pain, always increased by taking a full breath; moving about also excited it, and exercise aggravated its severity, until at length she was obliged to stop.

7th. To-day, for the first time, I injected the great intestine. About ten days since the pain in the left side very acute. I applied the second blister: it was kept open, but was now healed: it entirely relieved the pain. The woman was to-day flurried and frightened at the idea of an operation, and gave an indistinct account of her feelings. I visited her, however, in the evening, and then clearly ascertained that as the warm water flowed into the bowel, the first sensation felt was in the seat of the old pain; but it was not pain (she said), but a comfortable feeling of warmth, or "working," in the part. As the quantity increased to fulness, the usual gripings came on in the other parts of the bowel, to a very painful degree, but not the least in the seat of the old pain. I inquired if it occurred to her at this time, that the spot in which she formerly felt the pain was the same that now felt warm and comfortable. She said it was, and that at one moment she was about to mention it to me. Four pints of warm water were slowly injected, and retained half an hour; and the operation was repeated in the same manner every subsequent morning.

17th. The operation was repeated daily, with precisely the same feelings as at first. On making the experiment, she now found herself able to take exercise more freely than before, without increasing the pain in the side.

24th. The great intestine was daily injected in this case for a fortnight, and the operation then discontinued. The bowels, left entirely to themselves, were now relieved daily without medicine, having recovered the power of spontaneous action; neither was there any return of pain in the side under exercise. Having thus lost her complaint, she was allowed to go out, and has since remained well.

July 22, 1830. I examined the body of J. B. æt. fifty-six, who had died in the Infirmary from chronic diarrhœa, supposed to have arisen from ulcerated bowels. He had been five months complaining, but only of irritation, and a constant tendency to looseness. At first his bowels were disturbed almost every hour, but by the assistance of medicines, with leeches and blisters to the abdomen, he was relieved; so that latterly his bowels were moved only two or three times in the day.

There was no abdominal tenderness till a few days before his death, when seized with rigors. He next day complained of pain in the lower part of the right side, with shortness of breath, and febrile heat, &c. He was bled, leeched, and blistered, and ordered medicines, without relief, and in three or four days died.

On opening the body, the cause of death proved to be acute inflammation in the lower part of the chest and lungs, upon the right side. Extensive adhesion, with a copious effusion of serum and fibrinous matter, were found between the lungs, the diaphragm, and ribs. I found no ulceration, or other disease of the intestines. The whole of the colon, however, was considerably enlarged, down to a part of the rectum, about seven inches above the sphincter, where, for the extent of one and a half inches, it was so closely contracted as to be nearly impervious. It just allowed fluid fæces, as I afterwards found, to flow through it, in a stream equal in diameter to a small goosequill.

Very desirous to preserve this appearance, I took great care not to pull or disturb it. I therefore tied the rectum low down as possible, dividing it below the ligature. I placed a second ligature on the middle of the colon, dividing it above, and separating its attachments to the spine, lifted out the detached part of the intestine upon both my hands into a large basin of cold water.

I next opened the lower end, washed out its contents, filled it with a strong solution of alum, in water, and tied it up again. Without lifting it out of the water, the upper end was now opened, washed out, filled with proof spirit, and tied up; and then, having brought the glass, filled with rectified spirit, with its top sloping to the edge of the basin, I gently raised the preparation on my hands, allowing the lower part to pass first into the glass with as little disturbance as possible.

Having allowed it to remain ten days in spirits, it was again placed in water, the ends cut off, the contents washed clean out, and the preparation again suspended for a month in fresh spirit, which was afterwards changed several times.

This preparation eventually exhibited a very clear and exact demonstration of what happens in permanent spasmodic contraction of the great intestine, the appearances in the parts remaining precisely as they were during life.

The internal opening of the stricture is seen brought into view by cutting away a part of the side of the intestine, admitting the passage of fluid matters only. The small aperture, exhibiting the mucous membrane puckered up into folds, by the contraction of the muscular fibres, surrounding it externally. The superior part of the bowel excessively distended, thin, and weak, from habitual and constant accumulation; the inferior portion only moderately and occasionally occupied, and of natural appearance and thickness.

Externally, the two longitudinal bands seen spread and dispersed over the enlarged part of the intestine above; moderately expanded over the part below, but so contracted as to be both drawn together into one very narrow band at the strictured part. This preparation, which is now in my collection, I shall be extremely happy to shew any gentleman who may feel desirous of seeing it.—*Med. Gazette.*

---

### *Observations on the Incubation of Morbific Germs.*
By Dr. George Gregory.

The study of the specific causes of fever may be dated from about the commencement of the last century. In the days of Sydenham, and prior to his time, pathologists were chiefly occupied with discussions concerning the proximate causes of fever, such as acidity of the juices, fermentation, putrefaction, or degeneration of one or more of the humors, &c. They described, indeed, with much accuracy, those direct causes of fever which are obvious to our senses, such as the variations of the atmosphere in respect to its heat and dryness, the qualities of the ingesta, irregularities of exercise, and deficient clothing. It was reserved, however, for Lancisi, Sir John Pringle, Huxham, Lind, Dr. Russell, Dr. Withering, and other eminent writers of the last century, to investigate those occult sources of fever, which have their origin either in the soil, or in the animal frame itself, and which we now designate as the infective or febrific germs. Throughout their writings many observations will be found upon the interval which elapses between the application of the morbific agent and the subsequent developement of symptoms; and it cannot be doubted but that, in all ages, this subject has been considered as deserving of attention. It does not appear, however, that these authors devoted themselves specifically to its investigation. For obvious reasons, little could be looked for concerning it from the writers of an earlier æra. But even in our own times, it has been comparatively neglected: I know, indeed, but of one author who has made it the subject of specific inquiry. In the fourth volume of the Dublin Hospital Reports, there is a paper by Dr. Marsh, entitled, Observations upon the Origin and Latent Period of Fever. In this essay Dr. Marsh has advanced many very profound and philosophical views regarding the origin of disease generally: and no one can rise from its perusal without a conviction of the advantages which may be expected from a more diligent attention than has hitherto been paid to the laws which regulate the interval between the reception and the developement of morbific germs. With reference to sanitary or police regulations, this subject is obviously of the highest importance. It assists also materially in the study of diagnosis; and it will be found to involve many questions of general interest, and not a few of a directly practical tendency.

That this branch of study has not hitherto been more diligently pursued, need not surprise us. To establish the many minute details which it requires, investigations must be entered into for that specific object, and continued for years with a degree of assiduity such as few men engaged in actual practice could afford to bestow. But it must also be confessed, that the experience of one individual, though devoted exclusively to this object, would be insufficient; and the glory of discovery, therefore, must be shared with many fellow labourers. Constituted as human nature is, this can hardly be expected, while so much remains to reward the unassisted labours of the ambitious pathologist.

Under this impression, I do not venture to offer myself as a guide to the profession, on a topic requiring such extended research, and such accurate and varied personal experience. My aim is only to make a beginning in so important a work, by contributing the few results of my own observation, and collecting the recorded opinions of others, so far as they are known to me. The experience of my contemporaries may serve to fill up some of the blanks which I may leave, while to other points, hitherto unexplored, the labours of posterity may be advantageously directed. One principal object, in fact, which I had in undertaking this task, was to direct the attention of observing physicians to this neglected branch of pathology, the ascertaining, with all reasonable accuracy, the usual periods of incubation of the various morbific agents or germs, as well as the anomalies which they present.

To the general subject of investigation I have given the title of the "Period of Incubation." This term was originally suggested by the French; and it appears well deserving of admission into the nomenclature of English medicine. The terms "latent" and "dormant periods," which will be found in most of our standard works, carry with them this objection, that they presuppose the absence of all symptoms during the incubative stage; a position which, though true in some cases, is far from being of general application. Indeed, the kind of symptoms present during the incubation of the several diseases propagated by infective germs, is a legitimate subject of inquiry. Dr. Marsh has several useful observations on this topic.

There is every reason to believe that the interval between the application of the exciting cause of a disease and the manifestation of its peculiar phenomena, must be liable to many variations. The causes of these variations, and the extent to which they go, are points which it is of great importance to determine. This subject has also been touched upon by Dr. Marsh, in the essay above referred to. "From numerous observations," he says, "I have been led to think that the latent period may be shortened, and the accession of constitutional symptoms accelerated, by the occurrence of what are technically called the exciting causes of disease." To illustrate this, he cites the following case: "A boy, twelve years old, was playing with a favorite dog, and was bitten on the nose: the injury was slight. A few days afterwards the dog died rabid. The wound soon healed, and the circumstance made no impression on the boy's mind, and was wholly forgotten. Four weeks afterwards, he was thrown by his companions into a ditch: he went home wet, chilled, and complaining that he felt ill. That very night, unequivocal symptoms of hydrophobia manifested themselves, which proved speedily fatal." In this case, the usual period of incubation of the hydrophobic germ was (probably in consequence of this accident,) anticipated by twelve days.

By a comparison of numerous cases with each other, the pathologist endeavours to ascertain the average periods, and the maximum and minimum periods. To the maximum period it is difficult to attach any precise limit, yet common sense teaches that some such must be set. Tales are gravely told of hydrophobia occurring twelve years after the infliction of the wound. These cases are undoubtedly fabulous: it may, indeed, reasonably be questioned whether any infective germ whatever can exist in the body twelve months, retaining the power of producing disease. The utmost limit to which my own experience, and that of the professional friends on whose accuracy I can rely, would warrant me in extending this period, is nine months. In the case of a lady, who died in Edinburgh of hydrophobia, under the care of the late Dr. Gregory, nine months elapsed between the bite and the developement of the disease. A physician of great eminence in this town related to me the case of an officer, who first suffered from severe ague nine months after leaving the island of Walcheren, the interval having been passed in the healthiest parts of London.

The diseases in which it appears of most importance to determine the periods of incubation of their respective germs are the following: 1. Ague and Remittent Fever; 2. Typhus; 3. Plague; 4. Epidemic Yellow Fever; 5. Cholera Spasmodica; 6. Smallpox; 7. Measles; 8. Scarlet Fever; 9. Puerperal Fever; 10. Hooping Cough; 11. Hydrophobia; 12. Gonorrhœa; 13. Syphilis.

I shall offer a few cursory reflections upon each of these topics of inquiry.

1. Ague and Remitting Fever. I am indebted to the kindness of Sir William Franklin, principal inspector of the medical department of the army, for the following document, illustrating the incubative period of Malarial or Endemic Fever.

"Notes relative to the Movement of a Detachment of De Roll's Regiment in Sicily, in the year 1810.

"On the 12th July, 1810, the regiment of De Roll marched from Milazzo, and encamped on the heights of Curcuraci, near Messina. A detachment was subsequently sent to occupy a large house called the Casa del Corso, at a short distance from the body of the regiment. This mansion is situate on an eminence, between which and the sea there is a swamp, distant from the house at least half a mile. The north and north-westerly winds, very prevalent in Sicily, blow, often very strongly, over this swamp, directly on the Casa del Corso. During the prevalence of these, no one ever slept there, or in the open air near the house, without suffering from intermittent or remittent fever."

This information Sir William Franklin obtained from a man who had managed the vineyard belonging to the farm for fifteen years, and who had himself contracted fever, from sleeping with open windows.

"At this house the troops took up their quarters on the 7th July, 1810. They consisted originally of eighty-three men, who were subsequently joined by eight others, making a total of ninety-one exposed to the germs of disease. On the 31st of July, (thirteen days after exposure to the malaria,) the first case of remitting fever was sent to hospital. On the 1st of August five others were reported. The following day the detachment moved from the Casa del Corso, and encamped near the rest of the regiment, which was then healthy: yet the men continued to drop, and the admission into hospital from this detachment took place in the following order:

| Number sent to hospital. | | Date of admission. | | Interval since exposed to malaria. |
|---|---|---|---|---|
| 1 | .. | July 31, 1810. | .. | 13 days. |
| 5 | .. | Aug. 1, | .. | 14 |
| 1 | .. | 2, | .. | 15 |
| 8 | .. | 3, | .. | 16 |
| 5 | .. | 4, | .. | 17 |
| 6 | .. | 5, | .. | 18 |
| 7 | .. | 6, | .. | 19 |
| 11* | .. | 7,* | .. | 20* |
| 12 | .. | 9, | .. | 22 |
| 4 | .. | 10, | .. | 23 |
| 8 | .. | 11, | .. | 24 |
| 2 | .. | 12, | .. | 35 |
| 3 | .. | 13, | .. | 26 |
| 1 | . | 15, | .. | 28 |
| 2 | .. | 20, | .. | 33 |
| 1 | .. | 26, | .. | 39 |
| Total, 77 men. | | 27 days. | | |

Strength of the detachment, 91."

From this return it appears that the average period of incubation of the paludal febrific miasm is twenty days, the minimum thirteen, and the maximum thirty-nine. Of the whole number exposed (for a fortnight) to the action of the morbific germ, fourteen only escaped an attack of the disease. Whether a further exposure would have brought those fourteen men under the influence of fever, must remain a matter of doubt. I am inclined, however, to think not, and that the chief danger, in all cases, is from first exposure. As a proof of the intensity in which the poisonous effluvium existed in this instance, it may be remarked, that of the total number attacked (77,) twenty-three died, being in the exact ratio of thirty per cent. This is the usual rate of mortality in small-pox, and it approximates also to that of the Epidemic Cholera, as it has recently shewn itself in Northumberland.

Dr. Marsh remarks (p. 493,) that many of the Irish labourers who are employed during the harvest in the fenny counties of England, have their first fit of ague after their return from Ireland. This assertion is established by numerous cases of ague, which, at certain seasons of the year, are admitted into the ward of Steeven's Hospital. The period of incubation is thus extended to several months. The maximum I would venture to place at nine months.

2. Typhus Fever. Frequent as is the contagious typhus in this country, it is by no means easy to meet with cases so circumstanced as to offer determinate data for fixing the period of incubation of its infective germ. The commonly received opinion, I believe, is, that such period is subject to great variety. Dr. Haygarth, from his own observations, deduced the conclusion, that the minimum period was seven days, and the maximum seventy-two days. Dr. Bancroft's opinion coincides very nearly with this. From observations made on the hospital attendants, on occasion of the return of Sir John Moore's army from Corunna, he inferred, that thirteen days formed the minimum, and sixty-eight the maximum period. Sir W. Burnett, in his "Account of a Contagious Fever at Chatham," relates the history of a party of men belonging to the St. George, at Spithead, sent on the 3d July, 1811, to assist in navigating the Dolphin troop-ship, whose crew were affected with typhus. On the 10th January (seven days after exposure), fourteen cases of fever were sent to the hospital ship from the St. George, and many subsequently, up to the 21st January (the eighteenth from exposure), after which period no cases occurred.

* Average.

Dr. Marsh has made it one principal design of his paper to shew, that, in the Irish epidemics, the apparent period of incubation of the typhoid germ was very short indeed. In several instances which he relates, the febrile rigor succeeded so immediately to the application of the contagious effluvium, that it is scarcely possible to conceive that the germ of disease could have operated through the medium of the absorbents. He adopts, therefore, the notion of its direct agency on the sentient extremities of the nerves. An obvious objection, however, is open to all the cases cited by Dr. Marsh. During the prevalence of epidemic fevers so widely diffused as those adverted to by this author, there is every probability that the infective germ had previously been received into the system.

The following series of cases, which are not open to this source of fallacy, occurred under my own observation in the year 1819; and to those who have doubts on the subject, they may be adduced as undeniable proofs of the spreading of typhus fever by infection in private houses, with every advantage which ventilation, personal cleanliness, and all the comforts of domestic life, can afford. The cases were all seen by my friend Mr. Johnston, of Mortimer street, and they occurred in the family of a medical gentleman resident in that neighbourhood.

Case 1. A. B., maid servant, recently received into the family, sickened with typhus fever May 16, 1819. Died May 21.

Case 2. C. D., the mistress of the house (whose attention to her servant during her illness had been unremitting,) sickened with typhus May 24: died June 3, under the care of the late Dr. Latham.

Case 3. E. F., maid servant, who had attended A. B. during her illness, sickened with typhus May 30: came under my care June 7; died June 9.

Case 4. G. H., sister of A. B., residing in Clare market: she visited her sister during her illness; sickened with fever May 26; was delivered of a dead child June 2. June 11, seen by me, and found convalescent, though still in bed.

Case 5. J. J., cook in the same family, sickened with typhus May 29, and removed to her own home. I found her on the 10th June, convalescent, under the care of Mr. Smith, of Red Lion street.

The periods of incubation in these cases may be stated at seven, nine, twelve, and thirteen days from the first exposure to the infective germ. I have every reason to believe that ten days may be viewed as the average period in the case of the typhoid miasm.

3. Plague. From the concurrent testimony of numerous authors, we are warranted in saying, that the period of incubation of the true pestilential germ is very short. We may even lay it down as a maxim in the pathology of fever, that the more malignant the disease, the shorter is its period of incubation. Two has been stated as the minimum, and fifteen days as the maximum, period. Five days may be looked upon as a fair average.

When the plague has been received by inoculation, constitutional symptoms begin on the fourth day.

4. Epidemic Yellow Fever. I am not now in possession of any series of facts, by which to determine the period during which the infective germ of this disease lies dormant. From the inquiries, however, which I had formerly the opportunity of making, through the kindness of W. W. Fraser, Esq., inspector-general of hospitals, I was led to believe, that the interval between exposure to contagion and the developement of symptoms varies from two to ten days.

5. Cholera Spasmodica. The Central Board of Health have given the following as the results of their extended inquiries into the incubative period of this singular disorder.

1. Out of 171 cases of Spasmodic Cholera at Berlin, 159 occurred within five days from exposure to the infective germ.

2. At St. Petersburg, in the case where single exposure to infection was best ascertained, the period of incubation ranged between one and five days.

3. In the Austrian territory, according to the reports of the Genoese Medical Commission, it was observed "that those who have absorbed the germs of the disease were generally attacked before the third, and not later than the fourth day."

"6. SMALLPOX. At the Smallpox Hospital, abundant evidence has been afforded that the period of incubation is usually about twelve days. I select a few cases, in illustration.

CASE I. Mary Argent was discharged convalescent from the Smallpox Hospital July 6, 1830. She slept that night with her sister, Susan Argent, who sickened for the smallpox July 19th; the eruption shewed itself July 21st; she was admitted into the hospital July 22d. The period of incubation, counting (as I always recommend should be done) from exposure to the appearance of eruption, was in this case fifteen days.

CASES 2 and 3. Elizabeth Hall, aged three years, residing in Field place, St. John's-street, was attacked with smallpox October 25, 1829, and left that house for the hospital, October 28.

Henry Hall, aged five years (brother of the above,) was, after two days of fever, attacked with smallpox November 5, and received into the Smallpox Hospital November 7: period of incubation, eleven days.

Alfred Taylor, aged five years, living in the same house, was, after three days of fever, attacked with smallpox November 14: period of incubation (presuming that he took the disease from the second child), nine days.

CASES 4, 5, 6, and 7. Sarah Harman, seventeen months old, left Clerkenwell workhouse (having had the smallpox for five days) on January 28, 1828.

February 6. Another child in the same workhouse, after three days of fever, took smallpox: period of incubation, fourteen days.

February 7. Another child attacked with smallpox, after six days of fever: period of incubation, fifteen days.

February 8. A fourth child attacked, after two days of fever: period of incubation, sixteen days.

February 10. A fifth child attacked, after four days of initiatory fever: period of incubation, eighteen days.

All these cases were received into the Smallpox Hospital.

CASE 8. Elizabeth Foster was attached with smallpox November 26, 1830, after four days of fever. She was exposed to smallpox November 14, when her sister, Lucy Foster, sickened with it, and was sent to the Smallpox Hospital: period of incubation, twelve days.

CASE 9. A young medical friend, Mr. Tebbs, some years ago accompanied me to the Smallpox Hospital on a Thursday. On leaving the wards, he expressed to me his firm conviction (from the peculiar feelings he experienced at the moment,) that he had received the germ of smallpox. He soon after became languid, and his appetite fell off. On Saturday, in the ensuing week, rigors supervened, and two days afterwards the eruption of smallpox. In this case the period of incubation was twelve days.

In the London Medical Gazette, vol. iii. p. 282, a series of cases is given by Mr. Cæsar Hawkins, in which the period of incubation was very short, in none exceeding ten days. In one of them, rigors supervened within a few hours after exposure to contagion, and the eruption shewed itself on the third day, or, at furthest, at the very commencement of the fourth. This corresponds with what has sometimes been observed by Dr. Marsh, in regard to typhus. A feeling of disgust was experienced at the time, so strong that the gentleman dreamed of it the following night, and the impression frequently recurred to his mind.

The source of contagion was, in all these instances, a dead body brought into the Windmill-street theatre, for dissection. The body had been probably dead fourteen or sixteen days before two of the gentlemen came near it. Mr. Hawkins has justly remarked, that this occurrence of the disease in several persons, from exposure to the effluvia of a body so many days after death, is well deserving of notice, and seems to confirm the suspicion recently entertained with regard to the same liability in the pestilential cholera.

From the same series of cases we may learn, in regard to the origin of disease, how much depends upon the susceptibility of the recipient of the infective germ. One of the gentlemen who took the smallpox from this source had been in the habit of frequenting the Smallpox Hospital, and three months previously had made drawings of the disease from patients, both before and after death.

From the results of my own experience in smallpox, six days is the minimum period of incubation which I chould be inclined to admit: I would place the maximum period at twenty-one days.

It would be improper to pass over, without some comment, that peculiar sensation recorded in two of the preceding cases, as having been perceived at the moment of imbibing the infective germ. I have heard of the same thing in many other instances, and the patient often expresses himself as being frightened. Dr. Marsh has alluded to the circumstance of a highly disagreeable and peculiar odour, as characterizing the reception of the typhoid miasm, and founds upon it a train of ingenious reasoning concerning the modus operandi of the infective germ. I would beg leave to point attention also to the languor, loss of appetite, and impaired rest, which attended the incubation of the variolous germ in the same case. This sometimes proceeds to so great an extent, that, in the patient's judgment, six or seven days, instead of two, elapse between the attack of disease and the appearance of eruption. Hence arises the necessity of counting the period of incubation, from the reception of the germ, to the occurrence of eruption.

It is worthy of remark, that, of the numerous cases received into the Smallpox Hospital, not one in twenty is able to trace the disease to any source of infection; but it is believed by the patient to arise from cold, fatigue, change of air, or some similar circumstance. While so much difficulty is thus experienced in tracing the origin of a well-known disease, which spreads, I believe, exclusively by infective germs, it need not create surprise if we encounter similar difficulties in developing the origin of a disorder so little known to us as the spasmodic cholera.

When the smallpox is received into the system by inoculation, seven days elapse between the insertion of the virus and the establishment of the fever. In this case, the appearance of eruption is contemporaneous with that of the fever. An opinion has long prevailed, that, from the difference of the incubative periods, the inoculated smallpox would take precedence of the natural disease. I believe this notion to be destitute of all foundation. My reason for saying so is, that, with very few exceptions, it will be found, when smallpox is "in the blood," thet vaccination will not advance.

7. Measles. My own experience furnishes me with no precise data for determining the period of incubation of the rubeolous germ. It is, I believe, generally understood to vary from eight to fifteen days, counting from the reception of the germ to the first attack of rigor. Dr. Bateman says, "from ten to fifteen days." But as the catarrhal fever ought undoubtedly to be viewed as constituting part of the incubative stage, so this period will be found to extend, in many cases, to twenty-one days. Dr. Marsh relates the following case.* "A single exposure to measles took place on the 23d March. On the 3d April, the catarrhal symptoms began; on the 5th the eruption shewed itself." Here the full period of incubation was thirteen days.

8. Scarlet Fever. The latent period of this miasm has been the subject of frequent inquiry. Dr. Withering's words are, "I have repeatedly had reason to

* Dublin Hospital Reports, vol. iv. p. 495.

observe, that it is upon the third or fourth day after exposure to contagion that the patients begin to complain." Dr. Heberden extends the period of incubation to five days. Dr. Blackburn says it varies from four to six days. Dr. Willan gives six days as the maximum period.

Dr. Maton has described in the Transactions of the College of Physicians (vol. v. p. 161,) a peculiar variety of scarlet fever, in which the latent period varied from seventeen to twenty-six days; average, twenty-one days. The difference in the periods of incubation constitutes one of the strongest diagnostic marks between this and the common scarlet fever. Dr. Maton mentions the cases of eight members of one family attacked by this disease. They all terminated favorably.

9. Puerperal Fever. I have been given to understand that the late Dr. Gooch considered three days as the average period of incubation of the puerperal peritonitis spreading by contagion. This disease, when fatal, terminates (according to the same author,) for the most part, on the sixth day.

10. Hooping Cough. I have never been able to ascertain, by any facts, the period of incubation of the germ of this disease; nor do I know that any author has hitherto directed his attention to it.

11. Hydrophobia. Some years ago I made notes of a series of cases of hydrophobia (taken indiscriminately in the course of my reading,) with the express object of determining the minimum, average, and maximum periods of incubation of the hydrophobic virus. The cases were thirty-one in number. The following was the result.

| Case. | Period of Incubation. | Authority. |
|---|---|---|
| 1 .. | 21 days. | Mr. Gray, Duncan's Comment., vol. xii. |
| 2 .. | 26 .. | Dr. Dickson, Med. Obs. and Inq., vol. iii. |
| 3 .. | 31 .. | Marshall on Hydrophobia. |
| 4 .. | 35 .. | Dr. Chambers, St. George's Hospital. |
| 5 .. | 36 .. | Dr. Pinckard's Cases of Hydrophobia, (seen by myself.) |
| 6 .. | 36 .. | Dr. Plummer, Ed. Med. Essays, vol. vi. |
| 7 .. | 38 .. | Dr. Babington, Med. Comment., vol. i. |
| 8 .. | 38 .. | Mr. J. Scruton, Duncan's Com., vol. xvii. |
| 9 .. | 39 .. | Dr. Johnstone, Memoirs Med. Soc. of London, vol. i. |
| 10 .. | 39 .. | Attended by myself. Med. Chir. Trans., vol. xiii. |
| 11 .. | 40 .. | Mr. Sawrey, Marshall on Hydrophobia. |
| 12 .. | 40 .. | Dr. Lister, in Dr. Bardsley's Reports. |
| 13 .. | 40 .. | Dr. Munckley, College Trans., vol. ii. |
| 14 .. | 42 .. | Mr. Parkinson, Marshall on Hydrophobia. |
| 15(average) | 44 . | Dr. Pinckard's Cases of Hydrophobia. |
| 16 .. | 47 .. | Dr. Babington, Medical Records. |
| 17 .. | 60 .. | Dr. O'Donnell, Med. Comment., vol. ii. |
| 18 .. | 60 .. | Mr. R. Simmons, Med. Facts, vol. v. |
| 19 .. | 60 .. | Dr. Marcet, Med. Chir. Trans., vol. i. |
| 20 .. | 63 .. | Dr. Wavell, Medical Records. |
| 21 .. | 72 .. | Duncan's Commentaries, vol. xvii. |
| 22 .. | 73 .. | Dr. Pinckard, (case of W. Waters.) |
| 23 .. | 74 .. | Ditto, (case of W. Rogers.) |
| 24 .. | 77 .. | Mr. A. Battue, Duncan's Comm., vol. iii. |
| 25 .. | 3 months. | Dr. Satterley, College Transact., vol. iv. |
| 26 .. | 4 .. | Dr. Fothergill's Works. |
| 27 .. | 4 .. | Dr. Dickson, Med. Obs. and Inq., vol. iii. |
| 28 .. | 4 .. | Ditto, (two cases, bitten by the same dog.) |
| 29 .. | 8 .. | Acta Norimb. |
| 30 . | 9 .. | Dr. James Gregory, of Edinburgh. |
| 31 .. | 9 .. | Mr. Gaitskell, Memoirs Med. Soc. London, vol. v. |

From this table it appears that the average period of incubation of the hydrophobic germ is forty-five days; the minimum, twenty-one days; the maximum, nine months.

In the largest proportion of these cases (that is to say, twenty-four out of thirty-one,) death took place within three days from the manifestation of symptoms; six died between the fourth and seventh day; one, only, lived eight days.

From this document we may learn to distrust those alleged cases of hydrophobia occurring within three weeks from the infliction of the wound, and which ended favorably.

12. Gonorrhœa. According to Mr. John Hunter, the latent period of gonorrhœa is subject to as great variety as the diseases strictly constitutional. He believes that in some instances the complaint has shewn itself within a few hours from exposure to infection; but he acknowledges that the interval usually ranges between six and twelve days. He states the maximum period at six weeks. In almost all the cases which have fallen under my own observation, the urethra being previously healthy, the latent period was one week. How far we are justified in adopting John Hunter's notions regarding the maximum and minimum periods, I am not prepared to say.

13. Syphilis. The same distinguished author was of opinion that the interval between the application of the syphilitic poison and its primary effects, was very uncertain, but on the whole longer than in the case of gonorrhœa. He considered that the variations of the latent period depended, in some degree, upon the kind of structure implicated. In some instances he believes that chancres have appeared twenty-four hours from exposure to the virus. But he admitted of a latent period extending even to two months. Seven days is usually, and I believe justly, considered as the average period. My own experience in this disease is too limited to enable me to add any thing to the observations of John Hunter, on the extreme periods; and, unfortunately, I am not acquainted with the opinions of our best surgeons on these points.

The interval usually assigned for the developement of the secondary symptoms of syphilis is six weeks, corresponding closely with the latent period of the hydrophobic poison. Cases, indeed, have been related, wherein secondary syphilis never manifested itself until many years after the disappearance of primary symptoms. The authority on which such cases rest is undoubted, but they are always open to suspicion.—*Med. Gazette.*

